# RAB14 GTPase is essential for actin‐based asymmetric division during mouse oocyte maturation

**DOI:** 10.1111/cpr.13104

**Published:** 2021-07-29

**Authors:** Yuan‐Jing Zou, Meng‐Meng Shan, Hong‐Hui Wang, Zhen‐Nan Pan, Meng‐Hao Pan, Yi Xu, Jia‐Qian Ju, Shao‐Chen Sun

**Affiliations:** ^1^ College of Animal Science and Technology Nanjing Agricultural University Nanjing China; ^2^ WEGO Holding Company Limited Weihai China

**Keywords:** actin, meiosis, oocyte, Rab GTPase, spindle

## Abstract

**Objectives:**

RAB14 is a member of small GTPase RAB family which localizes at the endoplasmic reticulum (ER), Golgi apparatus and endosomal compartments. RAB14 acts as molecular switches that shift between a GDP‐bound inactive state and a GTP‐bound active state and regulates circulation of vesicles between the Golgi and endosomal compartments. In present study, we investigated the roles of RAB14 during oocyte meiotic maturation.

**Materials and methods:**

Microinjection with siRNA and exogenous mRNA for knock down and rescue, and immunofluorescence staining, Western blot and real‐time RT‐PCR were utilized for the study.

**Results:**

Our results showed that RAB14 localized in the cytoplasm and accumulated at the cortex during mouse oocyte maturation, and it was also enriched at the spindle periphery. Depletion of RAB14 did not affect polar body extrusion but caused large polar bodies, indicating the failure of asymmetric division. We found that absence of RAB14 did not affect spindle organization but caused the spindle migration defects, and this might be due to the regulation on cytoplasmic actin assembly via the ROCK‐cofilin signalling pathway. We also found that RAB14 depletion led to aberrant Golgi apparatus distribution. Exogenous Myc‐Rab14 mRNA supplement could significantly rescue these defects caused by Rab14 siRNA injection.

**Conclusions:**

Taken together, our results suggest that RAB14 affects ROCK‐cofilin pathway for actin‐based spindle migration and Golgi apparatus distribution during mouse oocyte meiotic maturation.

## Graphical abstract

RAB14 localized in the cytoplasm and accumulated at the cortex during mouse oocyte maturation, and it was also enriched at the spindle periphery. Depletion of RAB14 caused large polar bodies, indicating the failure of asymmetric division. Absence of RAB14 did not affect spindle organization but caused the spindle migration defects, and this might be due to the regulation on cytoplasmic actin assembly via the ROCK‐cofilin signalling pathway. We also found that RAB14 depletion led to aberrant Golgi apparatus distribution. Exogenous Myc‐Rab14 mRNA supplement could significantly rescue these defects caused by Rab14 siRNA injection. Our results suggest that RAB14 affects ROCK‐cofilin pathway for actin‐based spindle migration and Golgi apparatus distribution during mouse oocyte meiotic maturation.

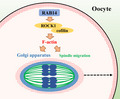

## 1 INTRODUCTION

For the cell division, the somatic cell is characterized by symmetric division, while mammalian oocyte is a unique asymmetric cell division. During the first meiotic division, with the segregation of homologous chromosomes, the oocyte divides asymmetrically which guarantees the retention of most maternal components within the oocyte.^1^ After germinal vesicle breakdown (GVBD), the meiotic spindle is organized in the central oocyte and then migrates along its long axis to the cortex of oocyte.^2^, ^3^ The chromosomes segregate near the oocyte cortex and a big oocyte and small polar body form.^4^ This process finally generates a highly polarized and metaphase II (MII)‐arrested oocyte, which is vital for fertilization and early embryo development. Spindle migration and positioning to the oocyte cortex are critical for correctly polar body extrusion which is accurately controlled by actin filaments,^5^ and these critical features ensure the oocyte asymmetric division during meiotic maturation.^6^

Actin nucleator Formin‐2 is required for the spindle migration to the cortex during the metaphase I (MI),^7^ while actin‐related protein ARP2/3 complex is another critical regulator for the oocyte asymmetric division.^8^ Besides actin nucleators, small GTPases are also involved in this process. RAN GTPase subfamily is shown to be essential for the formation of actin cap by activating two distinct pathways to regulate the localization and activation of ARP2/3 complex. The ARP2/3 complex can promote the formation of actin filaments flow from the cortical cap, and generate cytoplasmic streaming, which produce a pushing force for spindle migration to the cortex.^9^, ^10^ Besides, ADP‐ribosylation factor (ARF) GTPase subfamily member ARF1 mediates asymmetric cell division through regulation of MAPK activity in meiosis I,^11^ while ARF6 affects polar body extrusion by regulating actin filaments assembly through phosphorylating cofilin and inhibiting profilin activity in mouse oocyte meiosis.^12^ While Rho GTPase subfamily also plays a key role in mediating actin filament dynamics in oocytes. RhoA, a key member of Rho family, modulates the LIMK1/2‐cofilin pathway for actin filament distribution in oocytes.^13^ Our previous study also shows that the effector of Rho, Rho‐associated kinase (ROCK) participates in the actin filament assembly for spindle positioning in mouse oocytes.^14^

RAB GTPases are the largest subfamilies of the low‐molecular‐weight GTPase, which act as molecular switches that shift between a GDP‐bound inactive state and a GTP‐bound active state.^15^, ^16^, ^17^ Emerging data indicate that RAB proteins participate in vesicle budding, trafficking, docking and fusion.^18^, ^19^, ^20^ Moreover, RAB proteins promote the movement of vesicles on the cytoskeleton through its interaction with actin and microtubule motor proteins.^21^ Therefore, RAB GTPases are the central regulators of the pathway integrating vesicle transport for each step of events. Recent studies reported that RAB11A‐positive vesicles modulate actin network for asymmetric positioning of the meiotic spindle in mouse oocytes.^22^ Besides, RAB8A mediates ROCK for actin filament assembly, which is required for meiotic spindle migration and polar body extrusion.^23^ Similar with RAB8A, RAB35 facilitates the migration of spindle for oocyte asymmetric division via ROCK‐cofilin pathway during mouse oocyte maturation.^24^ These evidences indicate the potential roles of RAB GTPases on actin‐based oocyte asymmetric division.

RAB14 localizes to the endoplasmic reticulum (ER), Golgi apparatus and endosomal compartments, and it also localizes to the cleavage furrow and midbody during cytokinesis in human epidermal carcinoma cell line.^25^ Additionally, RAB14 GTPase plays a role in recycling pathways between the Golgi and endosomal compartments.^26^ Previous studies indicate that RAB14 regulates the trafficking of specific apical proteins from trans‐Golgi network (TGN) to apical early endosomal compartments in polarized epithelial cells^27^; and the overexpression of the GDP mutant form of RAB14 causes an increase in vesicle accumulation in the Golgi region and enlargement of TGN.^27^ In addition, RAB14 controls the translocation of GLUT4 from early endosome to the Golgi complex in 3T3 adipocytes.^28^ RAB14 also interacts with adaptor protein RAB11‐Fip2, which is class I RAB11 effector. This complex recruits the actin‐based motor protein myosin Vb, which tethers endosomal vesicles to the cortical actin cytoskeleton in epithelial cell.^29^, ^30^

Although previous studies reported the multiple roles of RAB14 in different cellular processes during mitosis, the functions of RAB14 during oocyte meiosis are not known. In present study, using mouse oocyte model we perform knock down and rescue approaches to explore the roles of RAB14 during oocyte meiotic maturation, and our results suggested that perturbation of RAB14 expression affected the actin filament assembly and spindle migration during mouse oocyte asymmetric division.

## 2 MATERIALS AND METHODS

### 2.1 Antibodies and chemicals

Mouse monoclonal anti‐Myc antibody (ab32), rabbit monoclonal anti‐ARP2 antibody (ab128934), rabbit anti‐N‐WASP monoclonal antibody (ab126626), rabbit monoclonal anti‐GM130 antibody (ab52649), rabbit monoclonal anti‐ROCK1 antibody (ab45171) (for western blot) and rabbit polyclonal anti‐RAB14 antibody (ab28639) were purchased from Abcam. A rabbit polyclonal anti‐ROCK1 antibody (bs‐1166R) (for immnuofluorescence staining) was purchased from Bioss. Mouse monoclonal anti‐α‐tubulin‐FITC antibody (F2168), the protease from *Streptomyces griseus* (P8811), phalloidin‐Atto 590 (93042) and Hoechst 33342 (bisBenzimide H 33342 trihydrochloride) (B2261) were obtained from Sigma. Rabbit monoclonal anti‐α‐tubulin antibody (#2125) and rabbit monoclonal phosphor‐cofilin (Ser3) antibody (#3313) were purchased from Cell Signaling Technology. Golgi‐Tracker Red (C1043) was obtained from Beyotime. Alexa Fluor 488 goat anti‐Rabbit antibody (ZF‐0511), and Alexa Fluor 594 goat anti‐Rabbit antibody (ZF‐0516) were purchased from Zhongshan Golden Bridge Biotechnology.

### 2.2 Collection and culture of mouse oocytes *in vitro*

All experimental procedures were performed according to the guidelines issued by the Animal Research Ethic Committee of Nanjing Agriculture University, and this study was specifically approved by the Animal Care and Use Committee of Nanjing Agriculture University. ICR mice (4‐6‐weeks‐old) were used to collect the oocytes. The mice were fed a regular diet and maintained at an appropriate temperature. The germinal vesicle stage (GV) oocytes of ICR mice were cultured in M16 medium surrounded by mineral oil at 37°C in a 5% CO_2_ atmosphere for *in vitro* maturation. Oocytes were used for different analyses at the appropriate culture time points.

### 2.3 Plasmid construction and mRNA synthesis

Ten ovaries were used to obtain RNA with an RNA Extraction Kit (Takara MiniBEST Universal RNA Extraction Kit), and the cDNA was generated by using the PrimeScript RT reagent Kit (Takara). The primers: F, 5′‐ACT AGT CCA GTG TGG TGG AAT GGC AAC TGC ACC GTA CAAC‐3′; R, 5′‐GCT GGA TAT CTG CAG AAT TCT AGC AGC CAC AGC CTT CTCT‐3′ were used to amplify the full‐length coding sequence of Rab14 by PCR. Purified PCR products were purified and cloned into MYC‐pcDNA3 vector with In‐Fusion HD Cloning Kit (Takara, Dalian, China). Rab14 mRNA was synthesized by using HiScribe T7 High Yield RNA Synthesis Kit (NEB), capped with m7G (5′) ppp (5′) G (NEB) and tailed with a Poly (A) Polymerase Tailing Kit (Epicentre), and then purified with RNA Clean & Concentrator (Zymo Research). Finally, the Rab14 mRNA was stored at −80°C.

### 2.4 Microinjection of Rab14 siRNA and mRNA

For RAB14 knockdown, Rab14 siRNA was microinjected into GV stage oocytes. Rab14 siRNA: 5′‐GCA CCG UAC AAC UAC UCU UTT‐3′ (Genepharma) was diluted with treated water to give a 50 μM stock solution. About 10 pl of siRNA solution was injected into GV oocytes. After that, the oocytes were arrested at the GV stage for 20‐24 hours in M16 medium containing 100 μM IBMX to facilitate the depletion of RAB14, followed by five times washes and transferred to fresh M16 medium for culture at the different time point. The control group was microinjected with the same amount of negative control siRNA. For protein expression and rescue experiments, GV oocytes were injected with 5‐10 pl of 800 ng/μL mRNA for the localization detection and 400 ng/μL mRNA for rescue analysis culturing in M16 medium with 100 μM IBMX for 2 hours, and then released in fresh M16 medium for further study. DNase/RNase‐free water microinjected as the control.

### 2.5 Real‐time quantitative PCR analysis

Total RNA was extracted from 50 oocytes using a Dynabeads mRNA DIRECT kit (Invitrogen Dynal AS), and the first‐strand cDNA was generated with the PrimeScript RT Master Mix (Takara), using the following conditions: 37°C for 15 minutes, 85°C for 5 seconds and 4°C. Rab14 cDNA fragments were amplified using the primers: F, 5′‐ATG GCA ACT GCA CCG TAC AA‐3′; R, 5′‐CTC CGT GTA ACC GCT CTGA‐3′ (GENEWIZ). Polymerase chain reaction (PCR) system was used with this condition: 30‐second denaturation at 95°C, 40 cycles of PCR for the quantitative analysis (95°C for 5 seconds and 60°C for 30 seconds), a melt‐curve analysis (95°C for 5 seconds, 60°C for 60 seconds, 95°C for 1 seconds), hold at 4°C. The fold change in Rab14 relative expression level was determined by comparison with GAPDH using the comparative 2‐ΔΔCt method. We performed at least three times per sample.

### 2.6 Immunofluorescence staining and confocal microscopy

The oocytes were fixed in 4% paraformaldehyde for 30 minutes at room temperature for antibody staining, and then transferred to phosphate‐buffered saline (PBS) supplemented with 0.5% Triton X‐100 for 20 minutes of permeabilization. The oocytes were blocked in PBS containing 1% bovine serum albumin at room temperature for 1 hour. The oocytes were incubated with anti‐Myc antibody (1:100), anti‐α‐tubulin‐FITC antibody (1:200), anti‐GM130 antibody (1:100) or anti‐ROCK1 antibody (1:100) at room temperature for 6 hours or overnight at 4°C. The oocytes were further incubated in the appropriate secondary antibody at room temperature for 1 hour. For actin staining, oocytes were stained with phalloidin‐Atto 590 at room temperature for 1 hour and then washed three times in wash buffer (PBS containing 0.1% Tween 20 and 0.01% Triton X‐100) for 3 minutes; each time. Hoechst 33342 was used to stain the chromosomes for 15 minutes. The samples were mounted on glass slides and observed under a confocal laser‐scanning microscope (Zeiss LSM 900 META, Zena).

We used live cell fluorescence staining to detect the distribution of Golgi apparatus. Protease was utilized to degrade the oocyte zona pellucida. A 10 mg/mL protease solution was aliquoted in M2 medium at a final concentration of 3 μM. MI stage oocytes were incubated in M2 medium and supplemented with protease in a 37°C atmosphere for 3 minutes, and then washed three times with M2 medium. Golgi‐Tracker Red was used to determine the distribution of Golgi in living oocytes. Golgi‐Tracker Red (1:100) was diluted in M2 medium at 4°C for 30 minutes; Hoechst 33342 was used for chromosome staining, and the oocytes were washed three times. The oocytes were incubated in M2 medium at 37°C for 30 minutes and immediately imaged under the confocal microscope.

### 2.7 Fluorescence intensity analysis

The control and treated oocytes were placed in different areas on the same glass slide for the fluorescence intensity calculation. The average fluorescence intensity per unit area within the region of interest was determined after fluorescence staining. The fluorescence intensity of the samples was analysed by ZEN lite 2012 and Image J software (National Institutes of Health, Bethesda, MD, USA). To quantify the fluorescence intensity, the confocal microscopy was performed in the same setting for the treatment and control oocytes.

### 2.8 Western blot analysis

Approximately 200 live mouse oocytes were lysed in LDS sample buffer at 100°C for 10 minutes and stored at −20°C. The treated samples were subjected to 10% sodium dodecyl sulphate–polyacrylamide gel electrophoresis, transferred to polyvinylidene fluoride membranes, and blocked in TBST containing 5% non‐fat milk for 1 hour at room temperature. The samples were incubated at 4°C overnight with primary antibodies against RAB14, ROCK1, ARP2, N‐WASP, α‐tubulin and phosphor‐cofilin (Ser3) (1:1000). Then, after washing three times for 10 minutes each in TBST, the membranes were incubated at 37°C for 1 hour with the appropriate secondary antibody. The membranes were washed three times with TBST, and the specific proteins were visualized using a chemiluminescence reagent (Millipore). Relative signal intensities were quantified using Image J software.

### 2.9 Statistical analysis

At least three biological replicates were used for each experiment. Moreover, each replication was used in an independent experiment at different times. Statistical analysis was performed by *t* test between control and treatment group. The experimental results are displayed as mean ± standard error. Data were evaluated using Graph Pad Prism 5 statistical software (Graph Pad Software). A *P*‐value <.05 was considered significant.

## 3 RESULTS

### 3.1 Expression and localization of RAB14 during oocyte maturation

**FIGURE 1:**
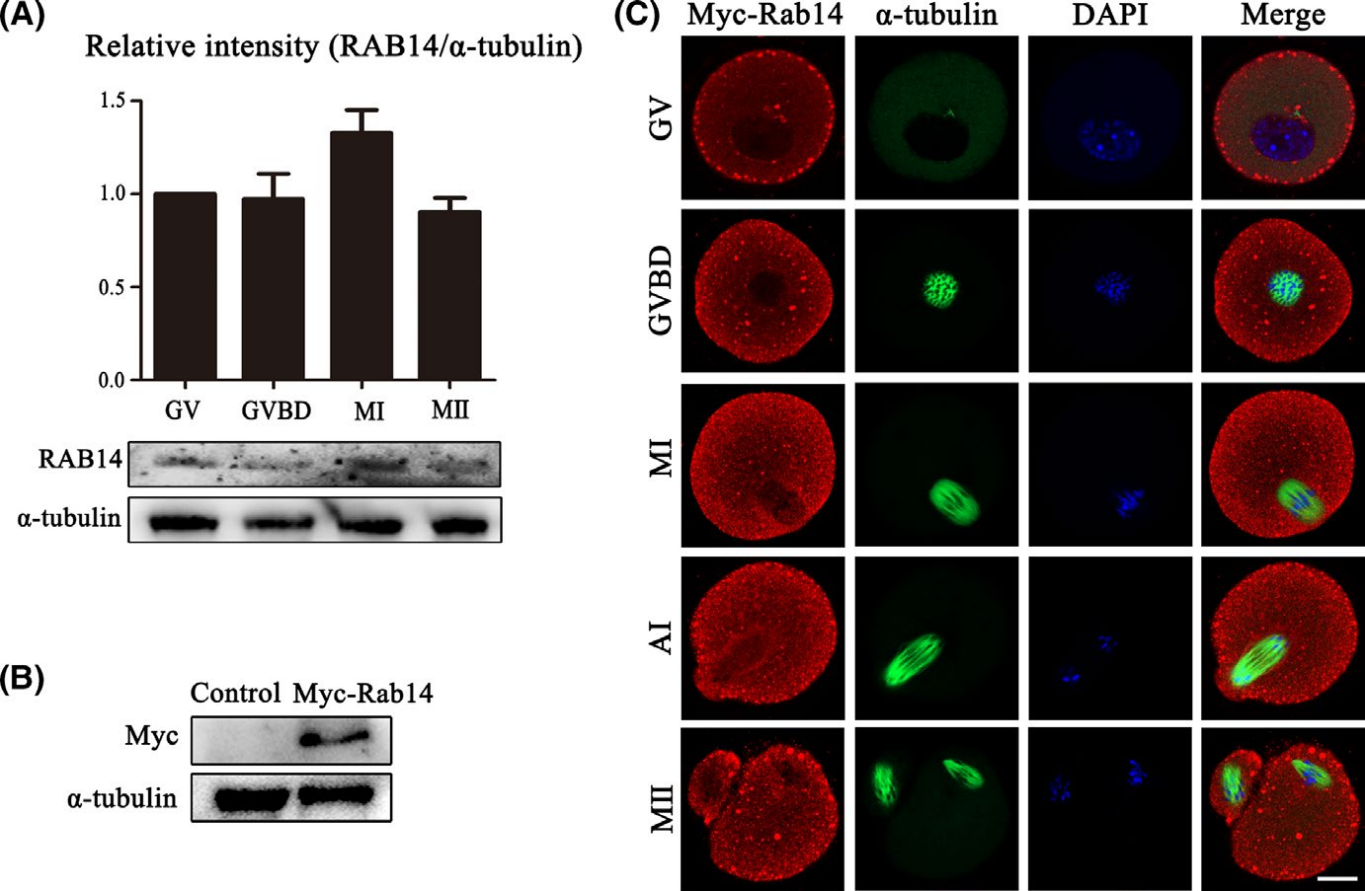
Expression and localization of RAB14 in mouse oocytes. (A) Mouse oocytes at different meiosis stages (GV, MI, ATI and MII) were examined by Western blot. Quantitative analysis of the relative intensity of RAB14 protein expression (relative to that of α‐tubulin) at different stages (GV, GVBD, MI and MII) indicated that RAB14 expressed during oocyte maturation. (B) Protein expression of exogenous RAB14 with an Myc tag was detected in the oocytes after the Myc‐Rab14 mRNA injection. (C) Oocytes from the GV to MII stages were stained with anti‐Myc antibody (red) and counterstained with Hoechest 33342 to visualize DNA (blue). The RAB14 protein was detected in the cytoplasm and cortex during oocyte maturation, and RAB14 was also accumulated at the spindle periphery after GVBD in mouse oocytes. Green, α‐tubulin. Bar = 20 μm

We collected mouse oocytes at the germinal vesicle (GV), germinal vesicle breakdown (GVBD), metaphase I (MI) and metaphase II (MII) stages, respectively. As shown in Figure 1A, RAB14 was stably expressed during all stages of mouse oocyte meiosis. Next, we injected Myc‐Rab14 mRNA into the GV stage oocytes to detect the subcellular localization pattern of RAB14 in oocytes. Our results showed that the Myc‐Rab14 protein expressed in oocytes after microinjection for 2 hours (Figure 1B). We cultured oocytes for 0, 4, 8, 10 and 12 hours to reach the GV, GVBD, MI, anaphase I (AI) and MII stages, respectively. As shown in Figure 1C, RAB14 localized in the cytoplasm and accumulated at the cortex from GV to MII stage as dots, and we also found the enrichment signals of RAB14 at the spindle periphery area during the MI, AI and MII stages after GVBD.

### 3.2 RAB14 ensures asymmetric division in mouse oocytes

**FIGURE 2:**
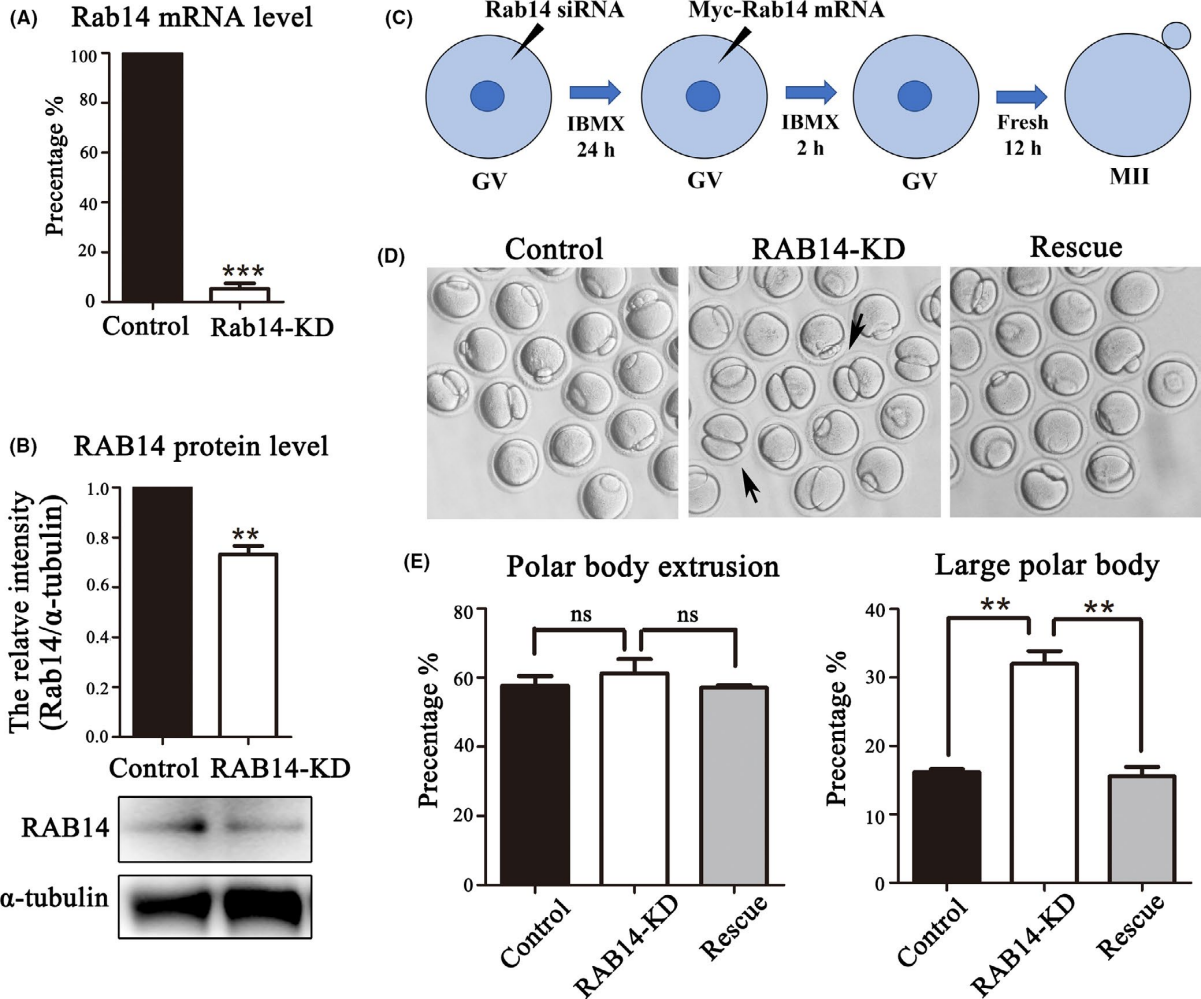
RAB14 knockdown affects asymmetric division in mouse oocytes. (A) Real‐time quantitative PCR results of the Rab14 mRNA expression level in the Rab14 siRNA‐injected group and the control group. ***, significant difference (*P* < .001). (B) Western blot analysis for RAB14 expression in the Rab14 siRNA‐injected group and control group. Relative intensity of RAB14 and α‐tubulin was assessed by densitometry. **, significant difference (*P* < .01). (C) Diagram for the rescue approach by Myc‐Rab14 mRNA microinjection after RAB14 knock down. (D) DIC images of control oocytes, RAB14‐KD oocytes and rescue oocytes after 12 h culture. RAB14 knock down caused large polar bodies in mouse oocytes (black arrows). (E) Rate of polar body extrusion and large polar body extrusion after 12 h culture of the control group, Rab14 siRNA‐injected group and the rescue group. No significant difference for the polar body extrusion between these three groups (*P* > .1). Rate of large polar body extrusion after 12 h culture increased in the Rab14 siRNA‐injected group compared with the control group and rescue group. **, significant difference (*P* < .01)

To investigate the functional roles of RAB14 during mouse oocyte meiotic maturation, we knocked down RAB14 protein expression via Rab14 siRNA injection. We collected oocytes of GV stage after microinjection for 24 hours to explore the knockdown efficiency of mRNA and protein levels. Real‐time quantitative PCR analysis showed that the Rab14 mRNA level significantly decreased after Rab14 RNAi (100% vs 8.78 ± 0.48%, *P* < .001, Figure 2A). We also observed a significant decrease of RAB14 protein level in RAB14‐KD group compared with the control group by Western blot and densitometry analysis (1 vs. 0.73 ± 0.03, *P* < .01, Figure 2B). Besides knock down approach, we also employed rescue approach by Myc‐Rab14 mRNA injection after Rab14 RNAi (Figure 2C). As shown in Figure 2D, A large proportion of oocytes exhibited large polar body extrusion (the polar body diameter was more than 1/3 of oocyte diameter) in the Rab14 depletion oocytes compared with the control oocytes in MII stage. The injection of Myc‐Rab14 mRNA rescued the defects of the large polar body extrusion caused by Rab14 RNAi. However, no difference was detected in polar body extrusion between the control and RAB14‐KD and rescue groups after 12 hours culture (control: 57.67 ± 2.85%, n = 162, *P* > .05 vs RAB14‐KD: 61.23 ± 4.11%, n = 175 vs rescue: 57.10 ± 0.72%, n = 157, *P* > .05, Figure 2E), while the rate of large polar body extrusion in the RAB14‐KD group was significantly higher compared with control group and rescue group (control: 16.18 ± 0.44%, n = 213, *P* < .01 vs RAB14‐KD: 32.03 ± 1.85%, n = 225 vs rescue: 15.58 ± 1.37%, n = 209, *P* < .01, Figure 2E). The results indicated that the depletion of RAB14 led to the defects of oocyte asymmetric division in mice.

### 3.3 RAB14 is essential for spindle migration in mouse oocytes

**FIGURE 3:**
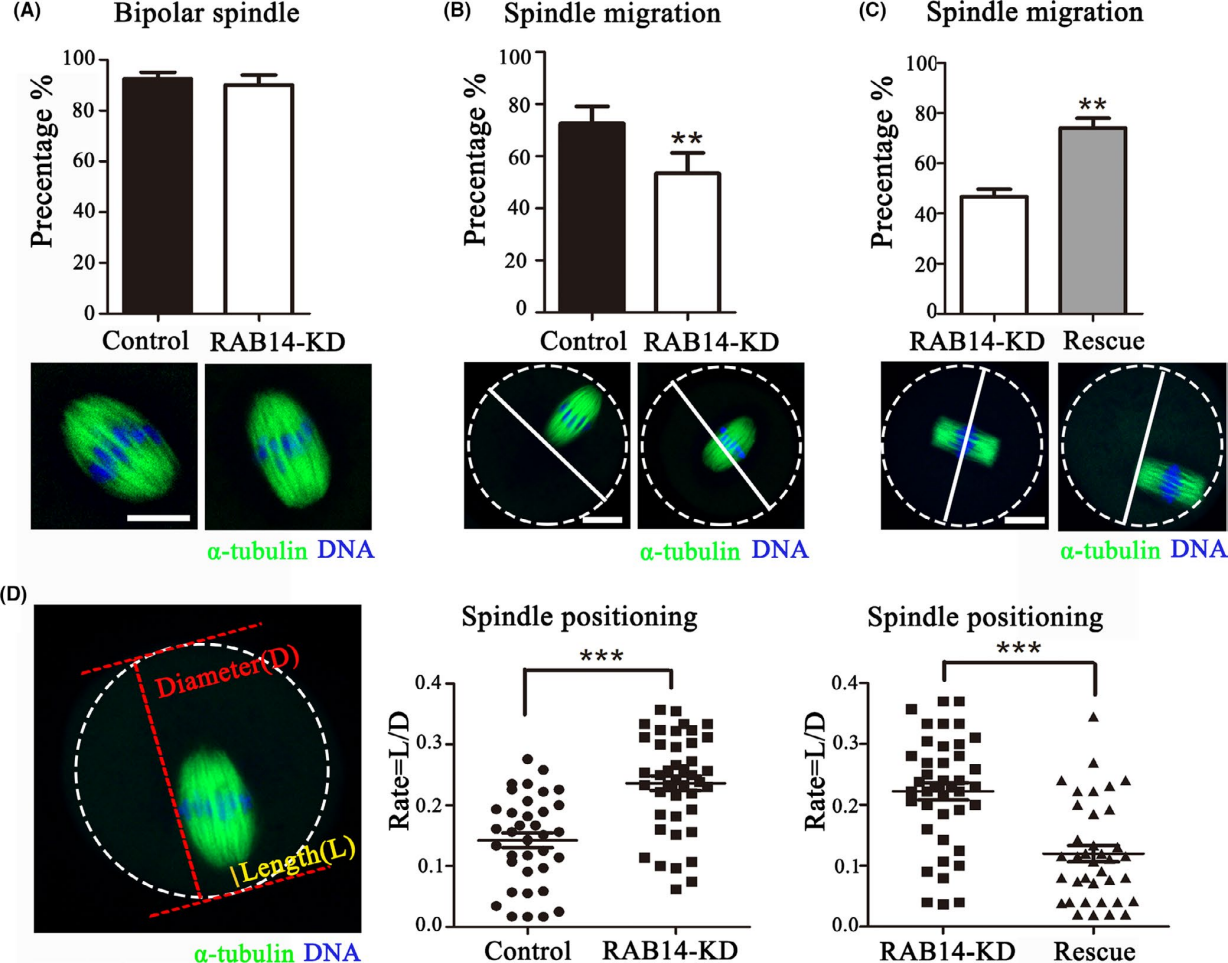
RAB14 knockdown impairs meiotic spindle positioning in mouse oocytes. (A) Representative images of spindle morphology (green) and chromosome alignment (blue) in the control and RAB14‐KD oocytes. No significant difference for the rate of bipolar spindle between these two groups (*P* > .1). Bar = 5 μm. (B) Representative images of the spindle positioning in the control and RAB14‐KD oocytes. Green, α‐tubulin; blue, DNA. Bar = 20 μm. The rate of spindle migration in the RAB14‐KD group decreased compared with the control group. **, significant difference (*P* < .01). (C) Representative images of the spindle positioning in RAB14‐KD and rescue oocytes. Green, α‐tubulin; blue, DNA. Bar = 20 μm. The rate of spindle migration increased in the rescue group compared with the RAB14‐KD group. **, significant difference (*P* < .01). (D) Calculation of spindle position to the cortex. The diameter of oocyte was defined as D, and the distance from spindle pole to oocyte cortex as L. The ratio of L/D increased in the Rab14 siRNA‐injected group compared with the control group, but decreased in the rescue group compared with the Rab14 siRNA‐injected group. ***, significant difference (*P* < .001)

Spindle migration during oocyte maturation is critical for polar body formation. To investigate the causes for large polar body, we examined spindle morphology and position after RAB14 depletion in MI stage. As the shown in Figure 3A, we found that RAB14 depletion did not affect spindle organization, and there was no significant difference between these two groups (96.24 ± 2.52%, n = 78 vs. 90.10 ± 3.94%, n = 86, *P* > .05). However, after 9 hours culture of oocytes, a large proportion of the spindles in RAB14‐KD group remained in the centre of the oocytes, whereas most spindles in the control group migrated to the cortex (72.50 ± 6.61%, n = 56, vs 53.37 ± 7.95%, n = 54, *P* < .01, Figure 3B). We also performed RAB14 rescue experiments, and the results showed that supplementing Myc‐Rab14 mRNA effectively rescued the defects of spindle migration compared with the RAB14‐KD group (46.70 ± 2.98%, n = 62 vs 74.13 ± 3.92%, n = 58, *P* < .01, Figure 3C). To confirm this, we quantified the distance between the spindle pole to the cortex (Length, L) and the diameter (D) of oocyte, to determine the cortically and centrally positioned spindles. Statistical analysis showed that the rate of the spindle migrated to the cortex (L/D) in RAB14‐KD group was higher than that in the control group (0.14 ± 0.01, n = 36, vs 0.24 ± 0.01, n = 42, *P* < .001, Figure 3D). In addition, asymmetric spindle positioning could be rescued by the microinjection of Myc‐Rab14 mRNA (0.22 ± 0.01, n = 41, vs 0.12 ± 0.01, n = 37, *P* < .001, Figure 3D). These data indicated that RAB14 mediated spindle migration for the asymmetric division of oocytes.

### 3.4 RAB14 involves into ROCK/cofilin‐based actin assembly in mouse oocytes

**FIGURE 4:**
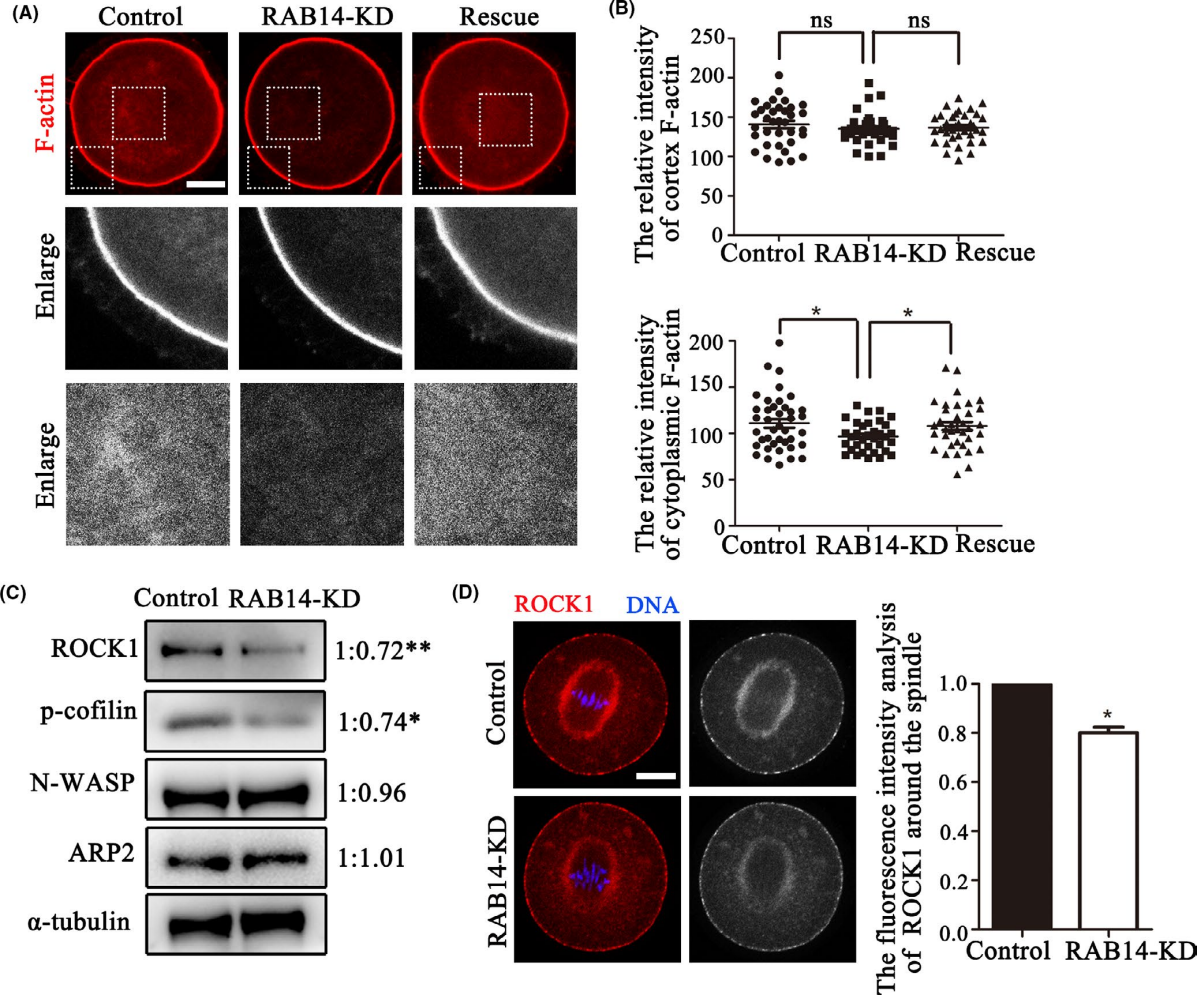
RAB14 knockdown disrupts cytoplasmic actin assembly in mouse oocytes. (A) Representative images of actin filament distribution at the oocyte cortex and cytoplasm in the control group, RAB14‐KD group and rescue group. Red, F‐actin. Bar = 20 μm. (B) No significant difference was observed between control oocytes, Rab14 siRNA‐injected oocytes and rescue oocytes for the F‐actin fluorescent intensities at the cortex (*P* > .1); however, F‐actin fluorescent intensities in the cytoplasm were decreased in the Rab14 siRNA‐injected oocytes compared with the control and rescue oocytes. *, significant difference (*P* < .05). (C) Quantitative analysis of the relative intensity of ARP2, N‐WASP, ROCK1 and p‐cofilin in oocytes by Western blot. The results indicated that ROCK1 and p‐cofilin protein expression were significantly reduced in oocytes after RAB14 depletion. **, significant difference (*P* < .01); *, significant difference (*P* < .05). (D) Representative images of ROCK1 in the control and RAB14‐KD oocytes. ROCK1 fluorescent intensities decreased in the RAB14‐KD oocytes compared with the control oocytes. *, significant difference (*P* < .05). Bar = 20 μm

To further explore the reason of the spindle positioning defects, we analysed the actin filament distribution after deleting RAB14 in MI stage. The results showed that there was no obvious difference in the accumulation of F‐actin fluorescent signals at the cortex between the control group, RAB14‐KD group and rescue group. However, cytoplasmic actin filaments of RAB14‐KD group were decreased compared with than that in control and rescue group (Figure 4A). The statistical analysis of F‐actin intensity also confirmed this finding: Cortex F‐actin: control: 140.71 ± 4.60, n = 35, *P* > .05 vs RAB14‐KD: 135.10 ± 3.41, n = 34 vs rescue: 136.62 ± 3.12, n = 36, *P* > .05; Cytoplasmic F‐actin: control: 110.85 ± 4.68, n = 40, *P* < .05 vs. RAB14‐KD: 96.65 ± 2.59, n = 36 vs rescue: 107.89 ± 4.32, n = 36, *P* < .05 (Figure 4B). We next investigated how RAB14 affected actin dynamics in oocyte meiosis. Western blot densitometry analysis showed that ROCK1 and p‐cofilin expression were reduced after treatment with Rab14 siRNA injection (ROCK1:1 vs 0.72 ± 0.03, *P* < .01; p‐cofilin: 1 vs 0.74 ± 0.05, *P* < .05); however, no change in the expression of the actin nucleators ARP2 (1 vs 1.01 ± 0.02, *P* > .05), N‐WASP (1 vs 0.97 ± 0.01, *P* > .05) (Figure 4C). Immunostaining results also showed that ROCK1 accumulated around the spindle in control oocytes, while the ROCK1 fluorescence intensity of Rab14‐depleted oocytes decreased compared with the control oocytes (1, n = 42 vs 0.80 ± 0.07, n = 46, *P* < .05, Figure 4D). These results indicated that RAB14 might affect ROCK‐cofilin signalling pathway for actin assembly in mouse oocytes.

### 3.5 RAB14 affects the distribution of Golgi apparatus in mouse oocytes

**FIGURE 5:**
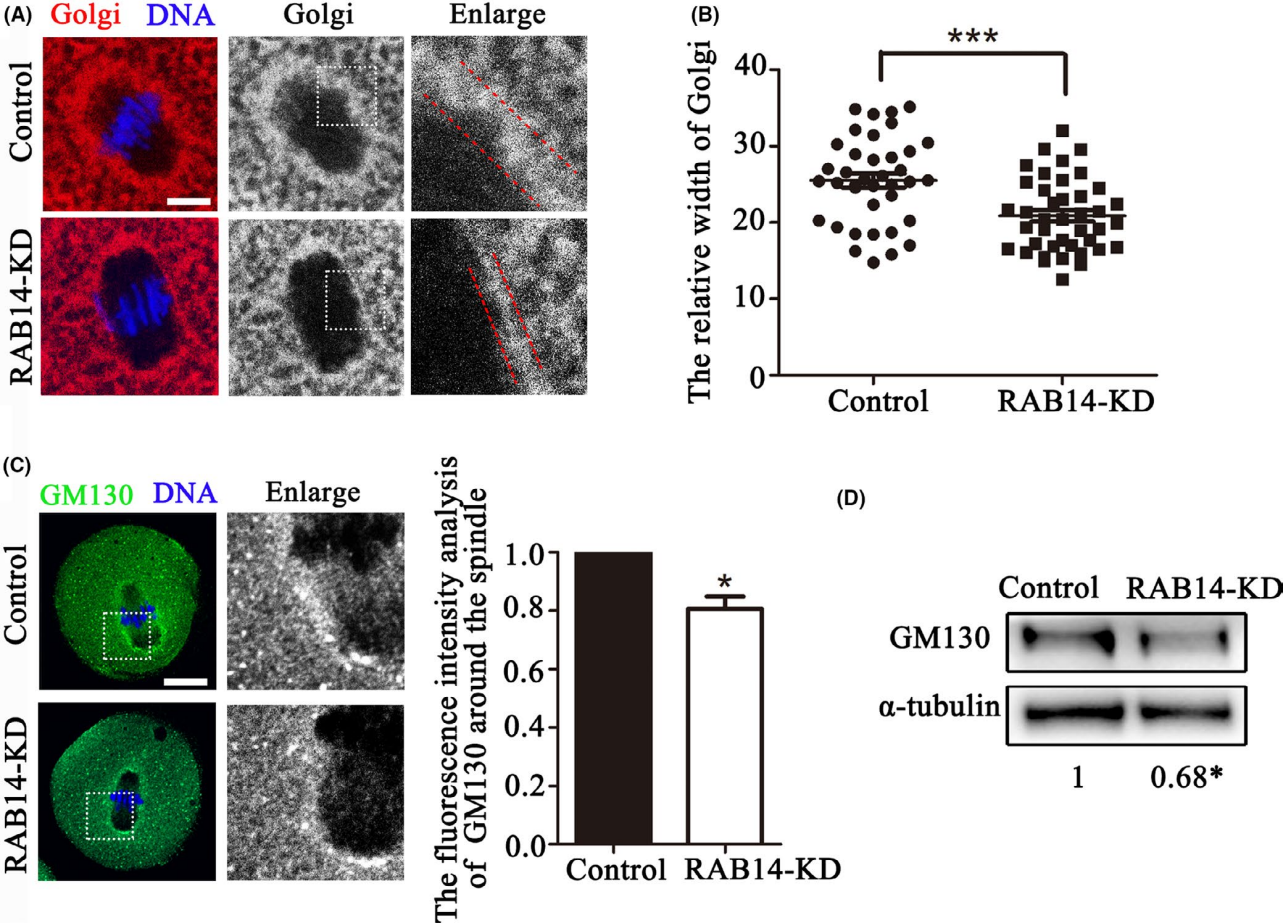
RAB14 knockdown affects Golgi apparatus distribution in mouse oocytes. (A) Representative images for the distribution of the Golgi apparatus in the control and RAB14‐KD oocytes. Red, Golgi; blue, DNA. Bar = 5 μm. (B) The relative width of the Golgi apparatus in the control and RAB14‐KD oocytes. ***, significant difference (*P* < .001). (C) Representative images of GM130 distribution in the control and RAB14‐KD oocytes. Green, GM130; blue, DNA. Bar = 20 μm. The fluorescence intensities of GM130 decreased in the RAB14‐KD oocytes. *, significant difference (*P* < .05). (D) Quantitative analysis of the relative intensity of GM130 protein expression (relative to that of α‐tubulin) in the control and RAB14‐KD oocytes. *, significant difference (*P* < .05)

The localization pattern of RAB14 at the spindle periphery was similar to that of the Golgi apparatus in oocytes, which prompted us to explore the potential role of RAB14 on the Golgi apparatus. The Golgi apparatus was mainly distributed around the spindle in the MI stage of control oocytes; however, the Golgi apparatus showed less accumulation at the spindle periphery area in the RAB14‐KD group (Figure 5A). We analysed the width of the Golgi‐Tracker signals at the spindle periphery, and the data showed that compared with the control group, the width of the Golgi‐Tracker signal in the RAB14‐KD group significantly reduced, indicating that Golgi apparatus structure was fragmented (25.51 ± 0.92, n = 39 vs 20.87 ± 0.74, n = 43, *P* < .001, Figure 5B). To confirm the relationship between RAB14 and the Golgi apparatus distribution in oocytes, we stained the oocytes with GM130, a cis‐Golgi marker. The results showed that GM130 accumulated at the spindle periphery and spindle poles in the control oocytes, while the fluorescence intensity analysis indicated that GM130 signal in the RAB14‐KD oocytes significantly decreased (1, n = 36 vs. 0.87 ± 0.04, n = 40, *P* < .05, Figure 5C). We next examined GM130 protein expression by Western blot analysis, and the results showed that GM130 protein expression was also significantly reduced after Rab14 RNAi (1 vs 0.68 ± 0.06, *P* < .05, Figure 5D). These results suggested that RAB14 was related with Golgi apparatus distribution in mouse oocytes.

## 4 DISCUSSION

**FIGURE 6:**
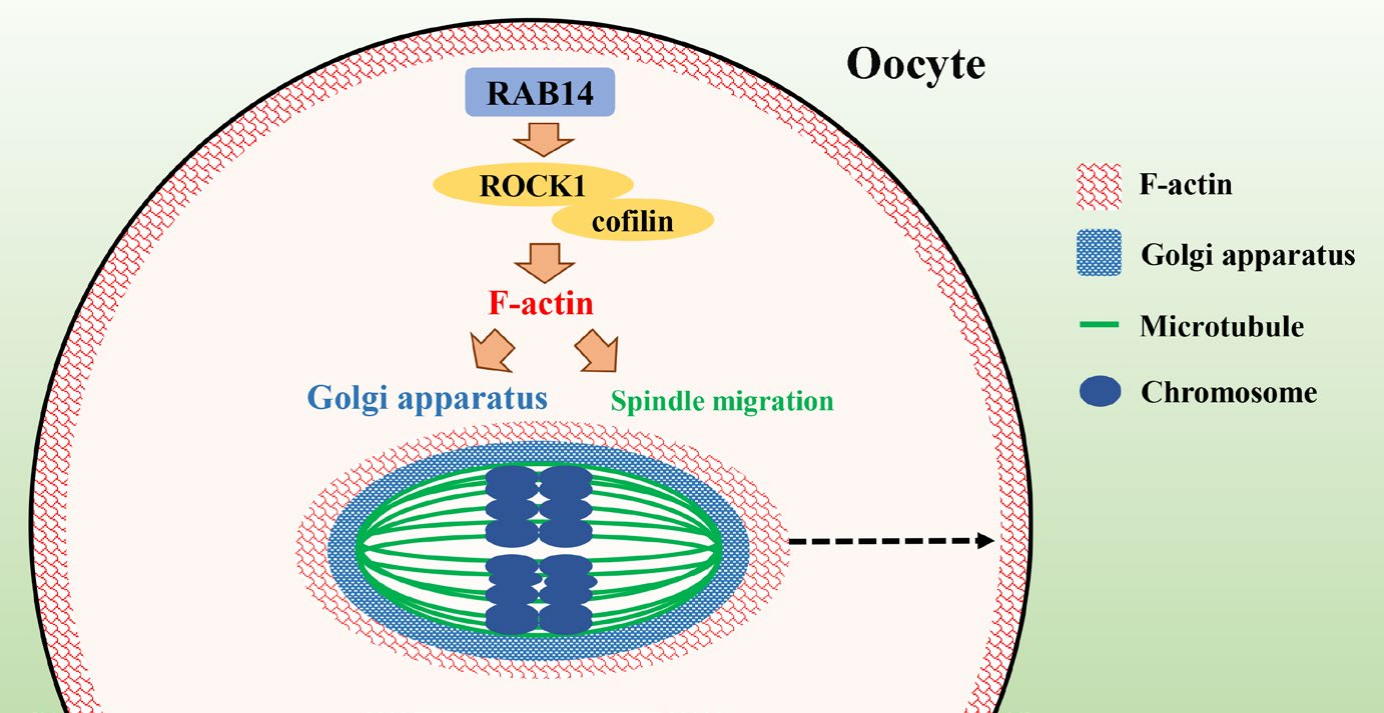
Diagram of RAB14 functions during oocyte meiosis. RAB14 plays a role in actin assembly, which might be through the ROCK‐cofilin signalling pathway for meiotic spindle migration and Golgi apparatus distribution in mouse oocyte meiosis

This study was designed to explore the potential roles of RAB14 during mouse oocyte meiotic maturation. Using knockdown and rescue approaches, we demonstrated that RAB14 affected Golgi apparatus distribution and spindle migration through its effects on ROCK/cofilin‐mediated actin assembly, which finally ensured asymmetric cell division in oocyte meiosis (Figure 6).

RAB14 is shown to localize at ER, Golgi and endosomal compartments in NRK cell, and cleavage furrow/midbody during cytokinesis in human epidermal carcinoma cell line.^25^, ^26^ Our results showed that RAB14 was continuously expressed during all meiotic stages of mammalian oocytes, and besides the cortex and cytoplasm localization pattern, RAB14 also accumulated around the spindle periphery at the metaphase, the area where ER and Golgi apparatus are located. The functional study results showed that the oocytes extruded large polar bodies after RAB14 depletion, indicating that RAB14 was critical for the asymmetric division. Due to the fact that the spindle relocates the position from the centre to the cortex of mammalian oocyte for the asymmetry,^31^ we then examined the spindle localization and the results indicated that RAB14 affected oocyte spindle positioning, which was responsible for the extrusion of large polar body. Similar findings were reported from other RAB family members in oocytes. RAB3A is shown to modulate peripheral spindle migration and polarity establishment for oocyte asymmetric division in mice, since RAB3A‐depleted oocytes display large polar bodies.^32^ RAB7 mediates actin dynamics for spindle migration, and perturbation of RAB7 also causes aberrant spindle migration and asymmetric division defects.^33^ Similar findings are reported for RAB24, since symmetrical eggs are frequently observed in RAB24 knock down oocytes.^34^

Dynamic meshwork of actin filaments plays a vital role in the asymmetric spindle positioning.^4^, ^35^ The interaction between RAB proteins and cytoskeleton is important not only for the regulation of intracellular transport but also in membrane and cytoskeleton remodelling.^36^ Recent study shows that RAB14 adaptor protein Fip2 binds to actin together with the actin‐based motor protein myosin Vb in *Chlamydia pneumoniae*.^37^ Our result indicated that RAB14 played a role in cytoplasmic actin assembly in mammalian oocytes. Several RAB family members are reported to participate in actin assembly during oocyte meiotic maturation. For example, RAB11A‐positive vesicles recruit the actin nucleation factors for actin assembly on the vesicle surface, and drive the network dynamics in a myosin‐Vb‐dependent manner, which is essential for asymmetric positioning of the meiotic spindle in mouse oocytes.^22^, ^38^ RAB35 plays a conserved role in the regulation of actin filament distribution and mediates actin‐based spindle migration in mouse oocyte meiosis.^24^ Similar phenotype is also reported for RAB6A since RAB6A‐depleted oocytes fail to form actin cap in oocyte meiosis.^39^ Our recent study shows that RAB23‐KIF17 cascade modulates the polarization and distribution of cytoplasmic actin filaments via RhoA signalling pathway and thereby regulates spindle migration during oocyte meiotic maturation.^40^ Our study indicated that RAB14 might regulate actin assembly through its effects on ROCK‐cofilin pathway in oocytes. Rho GTPase member RhoA is shown to participate in actin dynamics and regulate spindle positioning during asymmetric oocyte division in mice.^13^ As the effector of RhoA, ROCK participates in the formation of actin fibres and actin dynamics through the phosphorylation of cofilin, and then leads spindle migration during oocyte meiosis.^14^ It should be noted that besides RAB14, recently several RAB GTPases such as RAB35 and RAB23 are all shown to mediate RhoA‐ROCK‐cofilin for actin assembly in oocytes,^41^ whether these RABs interacts with each other for ROCK‐cofilin recruitment is still unknown. Moreover, due to the fact that ROCK is an effector of Rho GTPase, the relationship between Rho GTPase and RAB GTPase needs more investigation.

The Golgi apparatus plays a central role in intracellular protein modification and transport.^42^ Synthesized proteins are transferred to Golgi apparatus for glycosylation and other modifications, which support the development of oocytes.^43^ Moreover, the Golgi apparatus undergoes substantial re‐organization during meiosis, which is necessary for oocyte cytoplasmic maturation.^44^ While RAB14 is involved in the recycling pathway between the Golgi and endosomal compartments,^26^ our results showed that RAB14 affected the spindle periphery distribution of Golgi apparatus and GM130. GM130 is a cytoplasmic peripheral membrane protein, which localizes at the cis‐Golgi apparatus and plays roles on vesicle tethering and the structure of the Golgi apparatus.^45^ Absence of GM130 could induce aberrant Golgi apparatus organization and positioning.^46^ Similar findings in other RABs are also reported in previous studies. The distribution pattern of Golgi apparatus is shown to be regulated by RAB6, RAB41 and RAB30.^47^ In previous reports, the Golgi membrane and Golgi‐derived vesicles are shown to contain actin and actin‐binding proteins, which demonstrates that actin and Golgi might be interacted.^48^ The polymerization and dynamics of actin filaments could be used as a force for the scission, pulling and propelling of the transport carrier generated in cisternae, and for maintaining the structure of Golgi.^49^, ^50^ However, actin is necessary to maintain the structural integrity of the Golgi apparatus: interference with the actin machinery leads to fragmentation and dispersion of the Golgi apparatus.^51^ Moreover, RhoA‐ROCK‐cofilin signalling pathway is shown to regulate the formation of Golgi outposts in major dendrites.^52^ Rho family are key regulators of organelle actin‐driven events, and lacking the Rho‐binding activity will result in the dispersion of the Golgi apparatus.^53^ Therefore, RAB14 might alter the structure of Golgi apparatus and cause fragmentation through its effects on ROCK/cofilin‐based actin filaments in mouse oocyte meiosis.

In summary, our results indicate that RAB14 is essential for actin filament assembly, which may be through the ROCK–cofilin signalling pathway for meiotic spindle migration and Golgi apparatus structure during mouse oocyte maturation.

## CONFLICT OF INTEREST

The authors have no conflict of interest to declare.

## AUTHOR CONTRIBUTIONS

YJZ and SCS designed the study; YJZ performed the majority of experiments; YJZ, MMS, HHW and SCS analysed the data; ZNP, MHP, YX and JQJ contributed to the materials and reagents; YJZ and SCS wrote the manuscript. All authors gave final approval of the version to be published.

## Data Availability

All the original data including images from blotting and microscopy can be found in the manuscript and could be acquired from correspondence author.
